# Going virtual during the COVID-19 pandemic: adaptation of a mixed-methods dietary behavior study within a community-based participatory research study of African-American adults at risk for cardiovascular disease

**DOI:** 10.1186/s12874-022-01806-3

**Published:** 2022-12-22

**Authors:** Nicole Farmer, Ralph Thadeus Tuason, Narjis Kazmi, Sharon Flynn, Valerie Mitchell, Kimberly Middleton, Robert Cox, Kristina Franklin, Talya Gordon, Alyssa Baginski, Gwenyth R. Wallen

**Affiliations:** 1 Translational Biobehavioral and Health Disparities Branch, National Institutes of Health, Clinical Center, Bethesda, MD USA; 2 Social Determinants of Obesity and Cardiovascular Risk Laboratory, National Heart, Lung, and Blood Institute, Bethesda, MD USA

**Keywords:** Community-based participatory research, African-Amr, COVID-19, Virtual

## Abstract

**Background:**

Identifying mechanisms to maintain CBPR studies during an infectious disease pandemic is vital. The current paper describes the changes in methods and processes conducted within a CBPR mixed-methods study to a virtual setting during the novel coronavirus (COVID-19) pandemic.

**Method:**

The DC Community Organizing for Optimal Culinary Knowledge study with Heart (DC COOKS with Heart) was designed to assess the feasibility of a dietary behavior intervention among African-American adults that are at risk for cardiovascular disease (CVD). The study is under the umbrella of an ongoing CBPR study and community advisory board that facilitates community involvement in study design and promotes ongoing engagement with community members and leaders. The study population for D.C. COOKS with Heart consists of adult African-American individuals who live in two low-resource neighborhoods in Washington, D.C., which were impacted disproportionately by COVID. Eligible study participants who previously participated in the DC CHOC community-based studies were contacted to participate in Phase 1. The quantitative part of the mixed-methods included survey data collection.

**Results:**

Due to the pandemic, the mode of data collection for surveys changed from self-administered face-to-face to internet-based. All virtual study procedures were conducted between March and April, 2021. Anticipated benefits of the virtual setting included participant safety during the pandemic, ease of logistics for participants. Anticipated challenges included administration of electronic devices to participants, research team training, and potential threats to established trust related to the privacy and confidentiality of participants.

**Conclusion:**

The transition to a virtual setting for study procedures in a mixed-methods study was conducted successfully in terms of recruitment, retention of participants, and training of research team members. The virtual transition required established and ongoing engagement through the community advisory board and CBPR practices, institutional support through virtual research policies, collaborations with information technology-based teams, and equipment administration for the study.

**Trials registration:**

NCT04305431. Registered on March 12, 2020.

**Supplementary Information:**

The online version contains supplementary material available at 10.1186/s12874-022-01806-3.

## Background

The severe acute respiratory syndrome coronavirus 2 (SARS-CoV-2) virus emerged in late 2019 and had a consequential global impact. In March of 2020, the coronavirus disease-2019 (COVID-19) pandemic resulted in social and employment shutdowns, social distancing, and isolation. Early in the COVID-19 pandemic, communities of color, particularly, low-resource communities of color, were disproportionately affected in the United States [1]. The pandemic disrupted economies, educational systems, and ongoing research within communities, such as community-based participatory research (CBPR) studies. CBPR studies conduct research focused on addressing health factors germane to and derived from the community and thus involve the development of longitudinal relationships through repeated engagement with community members [2, 3].

Despite recent improvements in the global response to the pandemic, the ongoing presence of SARS-CoV-2 variants provides a potential for the continuation or reestablishment of social distancing practices that occurred early in the COVID-19 pandemic and that risked the continuity of CBPR studies. Ensuring the continuation of research conducted with communities, especially those disproportionately affected by health disparities during the pandemic, by adapting protocols to virtual settings is paramount to CBPR [4, 5]. For CBPR studies, the ability to participate in virtual research activities could positively influence recruitment, participation, and retention. For example, virtual research in CBPR studies could be beneficial by averting transportation and scheduling issues associated with in-person research study activities.

Although there are benefits to virtual research studies, there are potential disadvantages to consider. These include the security of the virtual platform, confidentiality and privacy within a participant’s home, and logistical needs of the participants in terms of equipment [6]. These potential disadvantages may be particularly important when conducting research with communities of color. For example, historical and ongoing mistrust of healthcare and research settings from minoritized communities such as African-Americans could lead to specific concerns about privacy and confidentiality when participating in virtual research.

The current paper describes the adaptation of methods and study processes conducted within a CBPR based study, The DC Community Organizing for Optimal Culinary Knowledge study with Heart (DC COOKS with Heart) in response to the COVID-19 pandemic. We present the experiences and methods of the research team with a focus on the roles of community engagement, institutional adaptation, research team resources, collaboration, and study budget planning. Although the research experience with converting in-person study procedures to virtual administration has been well-reported in the literature since the start of the pandemic [4, 5, 7, 8], little attention has been given to the experience of a CBPR study with a virtual study transition [9].

## Methods

### Original study protocol

The D.C. COOKS with Heart is a part of a long-term CBPR study to design culturally specific, community-based interventions to address cardiovascular risk factors, such as obesity. A community advisory board, the D.C. Cardiovascular Health and Obesity Collaborative (D.C. CHOC), was established in 2012 and is made up of a multidisciplinary research group, a university faculty in nutrition and community health, and church leaders from predominantly African-American, faith-based organizations in Washington, D.C. areas with the highest CVD prevalence and where access to physical activity resources and health nutrition is limited [10]. The Washington D.C. Cardiovascular Health and Needs Assessment (NCT 01927783) was the first research study designed by D.C. CHOC [11]. One of the overarching goals of the study was to assess potential psychosocial and environmental barriers to behavior change concerning physical activity and a healthy diet. Specific details of The Washington D.C. Cardiovascular Health and Needs Assessment can be found in prior publications from our group [11–13].

D.C. COOKS is a two-phase mixed-methods behavioral study designed to assess the acceptability and feasibility of a cooking behavior intervention among African-American adults that are at risk for CVD. A detailed description for both Phase 1 and Phase 2 of D.C. COOKS, including inclusion and exclusion criteria, is available in a previously published protocol paper [14]. Phase 1 includes the assessment of the acceptability of the intervention for the study population through focus groups and was adapted during the COVID- 19 pandemic. Phase 1 was thus designed to elicit findings from surveys (quantitative) and focus group discussions (qualitative) to identify potential barriers and facilitators to cooking behavior in the study population, as well as to inform the design of phase 2 [14].

In Phase 1, eligible study participants (*n* = 20) were to participate in one of the two moderated focus group sessions, held at a convenient community site. Focus groups were planned to have a moderator, co-moderators, and two note-takers present to record comments, as well as non- verbal observations. The focus group participants were to take electronic self-administered survey measures at the community site using research study tablets. The survey items used included questions related to cooking behavior, non-dietary health behaviors, and psychosocial factors [14].

Phase 2 of D.C. COOKS aims to determine the feasibility of a cooking intervention, especially in association with facilitators and barriers to cooking. It will also aim to explore the relationship between feasibility measures with intrapersonal, social, and built environment factors, dietary quality, and CVD biomarkers and anthropometric measures. Biomarker and anthropometric measurements are to be collected at baseline and follow-up clinic visits at the National Institutes of Health (NIH) Clinical Center in Bethesda, MD. Currently, in-person, virtual, or hybrid adaptations to Phase 2 are being considered due to the ongoing pandemic.

The original study protocol was approved by the (NIH) Intramural Institutional Review Board (IRB) in February 2020. After IRB approval, phase 1 was planned to start recruitment for focus groups utilizing a participant registry from The Washington D.C. Cardiovascular Health and Needs Assessment (NCT01927783) and from the DC CHOC sites. Using a purposive sampling approach, study staff were to contact and screen the potential participants based on eligibility criteria (African-Americans, age > 18, residents of ward 7 or 8, and self-reported risk of cardiovascular disease), with aims to recruit a diverse demographic group. The study procedures adhered to current Consolidated Standards of Reporting Trials (CONSORT) guidelines. Recruitment activities were planned, but was postponed due COVID-19 pandemic in March 2020.

### Study population and community engagement

The study population for D.C. COOKS consists of African-American adults (age ≥ 18) who reside within one of two neighborhoods (wards) in Washington, D.C.: Ward 7 or 8. Among the Washington DC population, these communities have the highest prevalence of CVD related risk factors and as defined by the United State Department of Agriculture are under-resourced in terms of accessibility to grocery stores/fresh food [15]. Home cooking frequency, a dietary behavior representing food and consumer choice, is positively associated with diet quality [16]. However, African-Americans, a population with a disproportionate burden of diet related disease, report less frequent home cooking on average compared to other racial/ethnic populations [16, 17]. Although cooking intervention studies in the African-American population show improvement in self-reported scores, home based cooking interventions may be required for sustainable behavior modification as participants acquire the knowledge and skills within the intervention related to nutrition education and that translates into dietary quality improvement [14]. As a result of the under-resourced food environments within Wards 7 and 8, these neighborhoods were selected for the D.C. COOKS study as the sites for intervention.

### COVID-19 pandemic restrictions and implications for D.C. COOKS

Wards 7 and 8 present significant economic and health disparities as compared to the rest of the city [1]. These neighborhoods, which represent the majority of the African-American population in the city [18], were impacted disproportionately by the pandemic, with higher mortality rates from COVID than other areas of the city. For instance, during the first year (2020-2021) of the pandemic African-Americans made up only 46% of the population in Washington, D.C. but consisted of 77% of the deaths from coronavirus in the city (https://coronavirus.dc.gov/data). In concert with CBPR principles of understanding ongoing and temporal concerns of a community, it was important that the study be made virtual not only to serve the needs of the research institution but also to protect and provide a safe, contactless experience for the study participants, whose daily lives were severely impacted by the COVID-19 pandemic. As a result of the challenges from the COVID-19 pandemic and the effects of the pandemic on the study community, the research team decided to make adaptations to the original protocol that would be required to provide virtual administration of the study procedures from Phase 1. Modification of the protocol included changes to the informed consent process, administration of electronic surveys, changes in the format of the focus groups, and to the focus group moderator guide. The changes to the protocol are discussed in the sections below including pertinent institutional roles, collaborations, and technology-based adaptations.

A tenet of CBPR is to establish long-term relationships with community members and leaders through multiple points of engagement. Following this tenet, participants in our prior CBPR study and members of the D.C. CHOC received updates quarterly on ongoing study activities through the distribution of a newsletter and CAB meetings. The D.C. CHOC coordinator and research team members also participated in community activities. During the COVID-19 pandemic, these community activities included virtual events. In August 2020 a presentation was made by one of the study investigators, who served on the Medical Executive Committee of the NIH CC, to the D.C. CHOC community board regarding the continuation of patient care and research through NIH policy modifications. In addition, the study PI provided an update to the D.C. CHOC on the plans to adapt Phase 1 of the study to virtual setting.

### Institutional role in adaptation

The research institution for D.C. COOKS is the National Institutes of Health Clinical Center. A single intramural Institutional Review Board serves the NIH Clinical Center for oversight of research conduct and human subject protections. An amendment was submitted to the NIH Intramural IRB in December 2020 to conduct the Phase 1 study protocol focus groups virtually. The amendment was approved by the IRB without stipulations in February 2021. The recruitment of participants started in February 2021. Virtual focus groups occurred between March to April 2021.

In addition to the IRB, departments within the NIH Clinical Center coordinated and collaborated with stakeholders from respective research study teams in implementing study procedures that align with federal policies regarding privacy and confidentiality. As a response to the COVID-19 pandemic, a majority of elective, non-acute, in-person visits shifted to telehealth visits for clinical research participants at the NIH Clinical Center. To ensure continuity of research and patient care activities, a telehealth policy was approved and implemented in April 2020. The telehealth platform approved was similar to a virtual teleconferencing platform. The approved telehealth platform was required to have enhanced security and privacy to meet the requirements of the NIH and had to meet protections to ensure Privacy Act compliance. The approved platform for our institution was Microsoft Teams, which was used to conduct the focus groups. A requirement by the NIH telehealth policy requirement was placing a telehealth appointment visit order (similar to placing an outpatient clinic visit order per NIH policy) in the patient’s electronic medical record. This step also prompts the NIH CC’s Telehealth support team to contact research participants to ensure that they have the appropriate device as well as provide the necessary technical support to complete the telehealth visit. The implementation of the platform led to a need for additional training and educational webinars for the research team. NIH Clinical Center Health Information Management Department provided the necessary training for us to successfully complete telehealth visits.

Prior to the COVID pandemic, the NIH Clinical Center Health Information Management Department initiated a pilot program of an electronic signature capture platform for signing informed consent forms. This platform can also be used to obtain informed consent signatures remotely and securely through a link sent through a text (SMS) or email. Our study team participated in the pilot program when the decision was made to transition the first phase of DC-COOKS to an approved telehealth virtual setting. The informed consent process obtained remotely was required to meet the same regulatory and policy requirements as an in-person consent process. The remote consent process reflected an in-person encounter as closely as possible. This included allowing for a real-time, verbal exchange of information between the consenting investigator and the participant to ensure the participant’s understanding of the research. Research staff who were allowed to obtain informed consent, as per the study protocol, underwent a one-hour training provided by the NIH Clinical Center Health Information Management Department on how to use the platform. The training was provided virtually and included synchronous training for available research team members, as well as asynchronous training for team members who could not attend.

### Technological adaptations: platform adjustments and testing

A perceived disadvantage of conducting qualitative research through a virtual setting is the loss of in-person non-verbal communication observations due to the limited face only view within virtual platforms. Due to pandemic restrictions, in-person options were not available with the study participants. Therefore, the research team sought to identify ways that in-person setting could be maintained between the moderator and co-moderator. The research team tested a technology platform that could potentially present the moderators and co-moderators of the focus group to be in the same room as in a traditional in-person setting. The platform was the Microsoft Surface Hub (50″ screen size). During the platform testing, the screen size was advantageous in clearly seeing all 4 participants in the perspective of the moderator and co- moderator compared to a laptop or traditional computer screen. However, there were significant barriers identified during the testing phase which precluded the use of this technology. One, an NIH Clinical Center requirement during the pandemic was if more than one person was in the same room, both individuals will have to wear a mask as well as maintain a distance of 6 ft. from each other. The social distancing requirement placed an additional need for a member of the research team to be able to pan and zoom (in or out) the camera between the moderator and co-moderator, which the platform’s compatible camera did not have the functionality. Without that functionality, it could present a difficulty for our research participants to focus on the moderator and co- moderator when they’re speaking. Two, having the moderator and co-moderators wear a mask during the virtual focus group could be considered a barrier in a focus group setting in where non-verbal cues like facial expressions can come into play. Three, verbal sound quality, with a mask over the speaker’s mouth, was compromised and could be a contributor to miscommunication. The research team concluded that using the traditional personal laptop or personal computer in their home office was the best method as all the research team member as well as the moderator and co-moderator would be able to attend without a mask and have the best view of their faces during the focus group.

To follow institutional security requirements, REDCap (Research Electronic Data Capture) housed in the NIH Clinical Center Biomedical Translational Research Information System (BTRIS) server was selected as a secure, web-based application for data collection of the electronic surveys [19].

In previous studies with the study population, smartphones were identified as the device of choice by participants when participating in studies that utilized electronic delivery of surveys [20]. Therefore, the current research team tested the interface of the REDCap survey on smartphones, as well as tablets, to simulate the participant experience. The display of questions and answer choices in smartphones was noted to be not user friendly due to difficulty viewing questions and all answer choices for a question. As display issues may increase participant burden due to difficulty in viewing survey questions, and potentially complicate data collection, tablets were therefore purchased and mailed to each of the 20 participants to use for the survey as well as to attend the virtual focus groups. A basic tablet stand was also provided to participants for participant comfort as each focus group lasted 2 h.

The research team selected an Android tablet that has a minimum screen size of 10 in. Based on pilot testing of the surveys as well as planning for the virtual focus group, a 10-in. tablet will provide the participants the best experience for survey completion as well as for the focus groups. Android tablets were selected because there is a wider selection of cost-effective 10-in. Android tablets in the market. Participants were encouraged to use the tablets to complete the online surveys and for the virtual focus groups.

### Informed consent process

**Fig. 1:**
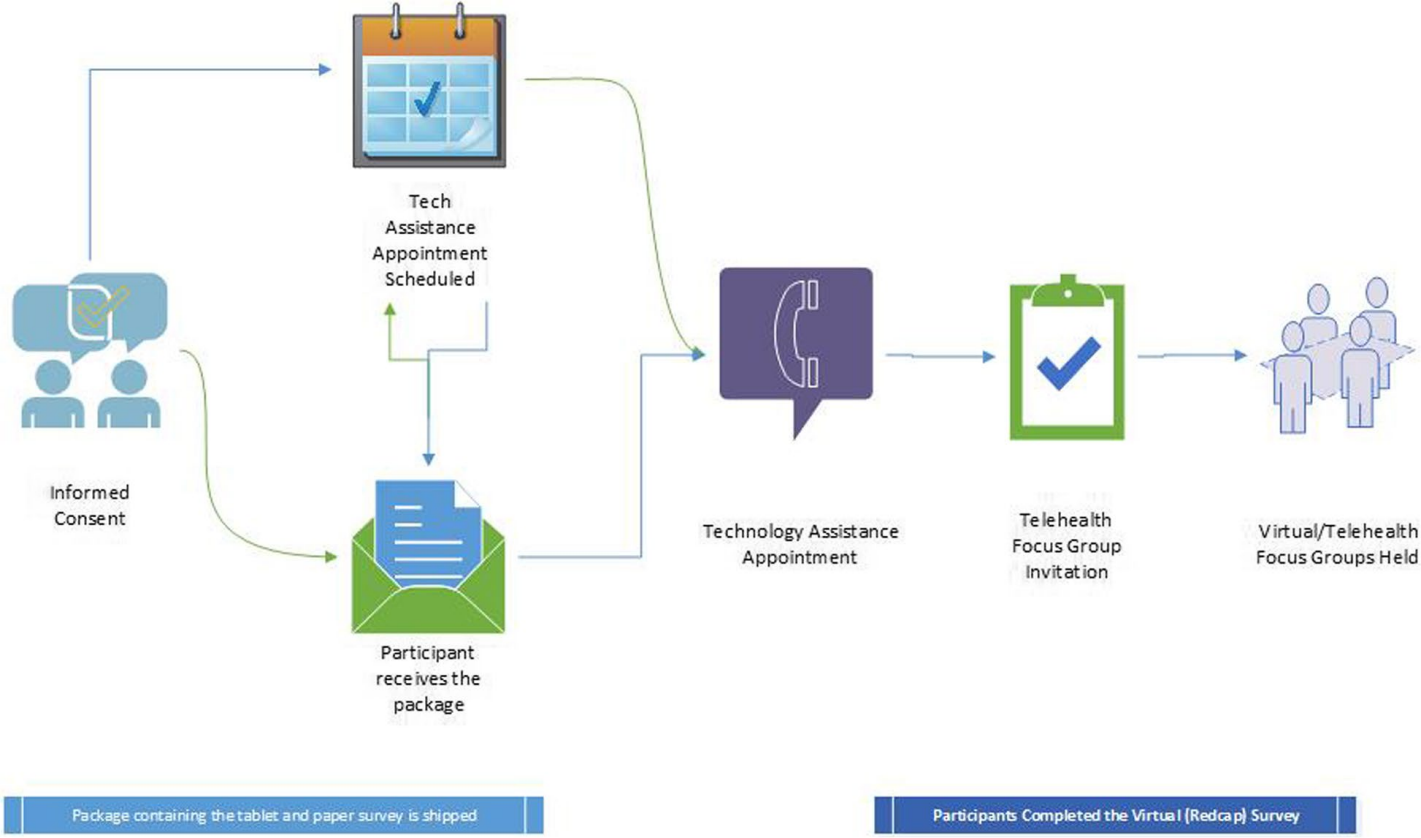
Study processes completed for informed consent, technical assistance to participants and electronic survey completion as a result of adaptation to virtual setting

Once a participant was screened and deemed eligible to participate in Phase 1, a packet including a copy of the informed consent containing the description of the study purpose and risks were mailed to the participant in advance of a scheduled phone appointment to review and obtain the informed consent (Fig. 1). During the scheduled consent phone appointment, the consenting investigator verified the participant’s name and date of birth, reviewed the consent in detail and the research participant provided verbal confirmation of understanding key elements of the study, as well as risks and benefits. The participant was then provided a secure link either by email or a SMS message to provide their electronic signature. The electronic signature capture was done synchronously in which an investigator obtaining informed consent was remotely present (via telehealth or telephone) while the participant was registering their signature electronically using the platform. After obtaining the electronic signature, the signed informed consent forms were securely transmitted and electronically archived into the research participant’s NIH Clinical Center medical record. A study team member mailed participants a copy of their signed informed consent forms. Utilization of the electronic consent was offered to all eligible participants and accepted by all participants except two. The benefit of the electronic platform avoided the delays and risks (loss) of mailing paper informed consent forms to and from research participants. The platform additionally minimized the need for participants to leave their home, which during the time of pandemic, helped to ensure participant safety. In terms of participant burden, despite admitting that they are technologically challenged, the majority of participants reported to research team members that the electronic informed consent process was easy to use especially when live assistance was available throughout the process.

### Quantitative data collection

Consented participants were mailed a paper copy of all survey questions to use during the focus group as a reference during the focus group discussions about survey questions. The online survey was “pinned” on the home screen of the study tablets provided. As a backup, the cover page contained the QR code and the website link that they can use to initiate and complete the surveys. The cover page also contained the participants’ study ID number to be used/entered for the surveys. Participants were asked to complete all survey questions prior to their scheduled focus group.

Advantages of changing to an electronically delivered survey included the ability for participants to take the survey based on their own schedule and time availability, as opposed to just prior to the focus group as planned when Phase 1 was planned in person. Disadvantages of the electronically delivered survey included the additional team resource to provide and mail a paper copy of the survey to participants, the time from the research team to assist participants with technical issues related survey administration (restarting/resuming surveys) on the provided tablet, and ensuring that participants completed the survey prior to the start of their scheduled focus group.

### Qualitative data collection

The initial in-person focus groups were planned to divide the study sample into two focus groups using a single moderator with each group consisting of 10 participants (total *n* = 20). As noted in the literature [5], virtual focus groups, ideally, call for a lower number of participants. An ideal number per group is 3-5 participants. We chose to have 4 participants in each focus group to maintain the total sample of 20. The principal reason for the lower number is to minimize difficulties that may arise with conducting a virtual focus group discussion with a larger number of participants.

All focus groups were scheduled to occur using the approved telehealth platform. The telehealth platform app was downloaded into the tablet prior to mailing it to participants. Participants were sent a secure email invitation from the study coordinator with the telehealth platform invite link to join their respectively scheduled focus groups. In addition to the NIH CC’s Telehealth support team contact, a research staff member also contacted the enrolled participants to provide additional technical support as needed. The frequency of phone call contacts for technical support ranged from one to four phone calls per participant. Depending on the participants’ skill level, the technical support time provided ranged from 5 min to approximately 1 h. The technical support included signing up for a Google account, tablet setup, assistance locating the focus group invite, connecting to Wi-Fi, downloading an email app, and reinforcing instructions on how to use the telehealth platform.

## Results

In March and April of 2021, five virtual focus groups were conducted with four participants in each focus group. The focus group manual and research team member roles were changed for virtual administration. The moderator and co-moderator roles were maintained and principally involved leading participants through the focus group discussion and questions. The co- moderator for the virtual focus group also took on the role of scanning the virtual room for cues as to when other participants wanted to contribute or may have shown body language suggestive of having a response to a moderator question or to another participant’s comments. Changes to the team included the addition of an information technology (IT) team member to help participants and the team during each focus group. The IT research team member was specifically responsible for admitting and managing participants in a virtual waiting room, for muting participants when there was distracting noise, and assisting the participants with technical issues during the focus group. Additionally, two notetakers were added to the team as opposed to one for each prior planned group. Notetakers were tasked with noting facial expressions and visible body language of the participants during the focus group. Due to the two-dimensional nature of virtual focus groups and to the presence of multiple faces on a screen to view, it was felt that two notetakers would be needed for each group.

The changes to the manual included the addition of instructions to participants for using the basic audio and video functions on the platform, confidentiality instructions regarding presence of non-study participants within audible distance, and instructions for contacting the IT research team member if technical difficulty arose during the focus group. All participants were asked to keep their video monitors on unless they had to excuse themselves from the group. The focus group sessions were audio recorded using a stand-alone digital recorder for verbatim transcription.

All 20 participants participated in their scheduled focus groups. During the five focus groups, no participants were removed or lost from the focus groups due to technical problems. Minor technical problems did occur during each focus group including: need for participants to adjust tablet volume or video screen for optimal viewing by the moderators, and momentary loss of the shared screen when moderator shared content. All tech issues were resolved with assistance with by the IT research team member. All participants used their own home Wi-Fi for access to the focus groups, with the exception of one participant who used their smartphone hotspot for internet access. All participants used the provided tablets and stands for the focus groups except for one individual who used their personal laptop.

After each focus group, the research team conducted a debrief session regarding participant involvement and topics discussed during the focus group. At the end of the last focus group the debrief discussion included the research team’s observation of data saturation across the five groups in terms of repeated topics being presented by the participants. The observation was based on research team members previously observing in-person focus groups conducted within our CBPR studies, which involved participants from this same community. These team members reported that the discussion and participant involvement was similar to those observed in the in-person groups.

## Discussion

Our study aimed to describe our research team’s experience with the transition of an in-person mixed methods study acceptability CBPR study, involving focus groups and participant-administered surveys, to a virtual setting during the COVID-19 pandemic. Electronic surveys were administered and five focus groups were conducted with African-American adult participants ranging in age from 34 to 67 years of age, and who reside in low-resource neighborhoods. Using study-provided electronic devices, videoconferencing software, and institutional infrastructure that included electronic informed consent software capability, we were able to successfully conduct and transition all study procedures during the mid-phase of the COVID-19 pandemic when social distancing and community-specific lockdowns were still occurring.

This study demonstrated several benefits and challenges of transitioning and conducting a virtual mixed methods study. The benefits of conducting the virtual focus groups included ease of scheduling for participants who at the time were homebound due to the pandemic and due to caretaking duties. For example, the ability to schedule after-work hours as opposed to scheduling based on the availability of a community space. In addition, there is the comfort of being within their home while discussing a home-based health behavior, cooking, was noted by the research team as a benefit.

Notwithstanding the perceived benefits of transitioning to a virtual setting, there were several challenges that resulted from the virtual setting. For instance, there was a reduction in the number of participants per focus group to allow to facilitate participation and moderator observations of participants. The reduction in participants per group led to an overall increase in the number of focus groups in order to reach data saturation. Other challenges included a lack of a controlled environment during the virtual focus groups. Within their home, participants may experience internet or technical difficulties or family members in the household may distract their attention.

Similar to others conducting virtual mixed-methods studies [21, 22], our study required the provision of electronic devices to participants. Providing electronic devices can assist in minimizing barriers to participation for participants with limited in-home wireless internet, and may prevent participants from using their own handheld devices which may have a personal expense through service carrier charges. As a mixed-methods study, we had another specific necessity for providing devices, to insure optimal and consistent visibility of the electronic surveys.

Technical issues interfering with focus group discussions and quality of recordings are noted challenges with virtual focus groups. Even with providing electronic devices for study procedures, technical difficulties were additional challenges that occurred during the focus groups [21–24]. Although our study participants were not exclusively of lower household socioeconomic status, all of our participants resided in a low-resource community with non-optimal internet access which presented an issue with connectivity. A lesson from this experience may be when planning virtual studies, it is not only important to consider participant-specific internet issues but also that of the community or locality. Despite our experience and that of others with technical difficulties during virtual focus groups [23, 24], comparisons between virtual and in-person groups have shown no differences in the quality of data obtained between the two approaches [4, 5, 25].

The administration of both the electronic surveys and virtual focus groups demonstrated the need for additional roles for the research team. Although specific to our institutional security requirements, the administration of electronic informed consent procedures and electronic surveys to participants via email led to intra-institution collaborations which required additional pieces of training for our research team. Team members also participated in pre-focus group technical support phone calls to ensure that the participants were comfortable with the use of the tablets and had access to the telehealth platform (videoconference software). Although this step required additional time from the research team, it may have fostered engagement with study participants, aligning with a core component CBPR partnerships in research [26, 27]. Conducting the virtual focus groups also required additional roles for team members, including an IT moderator role, and additional tasks for observers and note-takers, including monitoring the videoconferencing chat for comments or participants expressing technical issues.

Our findings are similar to recent literature on transitioning in-person studies to virtual during the COVID-19 pandemic. In a study with a population similar to our study’s, Lathan and Lituihain found that virtual focus groups were feasible to administer but unavoidable challenges related to technical difficulties, distractions within the home, and need for additional study costs such as electronic devices occurred. An additional consideration not found in our study but reported by others transitioning to virtual focus groups is the potential need to increase participant compensation [21, 28]. In-person focus groups often provide food or childcare opportunities, but in the virtual setting, the burden of providing food and childcare during the time of participation is shifted to the study participant.

There were several strengths within our study that may have aided our experience. Given the potential concerns of privacy and confidentiality with conducting virtual research, a higher threshold of established trust and rapport is required between the research team and community members in order for participants to consider study participation. As a study within a larger CBPR one, we were able to maintain continuity with the community, including community leaders and organizations, from which we recruit study participants. This continuity likely assisted our ability to recruit participants for a virtual study. Others have reported that recruitment for a virtual or remote study during the pandemic may be hampered by the lack of prior face-to-face contact [21] or require a potential shift to social media based recruitment [29]. Another strength was our institutional health informatics infrastructure which facilitated key elements of the transition to the virtual platform including electronic consent and secure videoconferencing software access.

## Conclusion

We anticipate that our future studies will continue to include virtual methods for electronic consent and focus groups. For future studies, we will have to plan for the additional staff time, potential costs for purchasing equipment required to conduct mixed-methods research virtually (Table 1). Our study’s experience was likely successful due to our prior engagement with the community as a CBPR study, as well as the institutional support in terms of our research institution implementing two key policy changes regarding electronic informed consent and telemedicine.

**Table 1:** Lessons Learned from transitioning a mixed-methods community-based dietary behavior study among African-American adults during the COVID-19 Pandemic

Lessons Learned	
**Community Engagement**	
• During an infectious disease pandemic, continuity of engagement with community members is feasible through utilization of virtual formatting	
• Planning for this type of engagement through providing education regarding equipment and selected virtual platforms for community members is necessary	
**Institutional Role**	
• Research institutions play a pivotal role in implementing policies and procedures that allow for utilization of institution services and resources	
**Roles and Training for Research Team Members**	
• Inclusion of virtual applications for research trainings and prioritization of trainings	
• Having diverse experiences and skills within the team to help team members in unforeseen circumstances (e.g. an IT team member)	
• Increased engagement with participants than in prior study plans to account for technology and equipment assistance	
**Role of Collaborations**	
• Collaborations with teams experienced in virtual or electronic research procedures can assist in filling in knowledge and skill gaps	
**Study and Budget Planning for Virtual Research Administration**	
• Establishing plans for pilot testing of technology	
• Forecasting budget costs for equipment or technology changes	
• For mixed-methods studies, it is important to conduct tandem planning for virtual administration of both qualitative and quantitative data collection	

## Supplementary Information

**Additional file 1.** Virtual focus group moderator’s guide for DC COOKS (2021).

## Data Availability

The datasets used and/or analyzed during the current study are available from the corresponding author on reasonable request.

## Abbreviations

BTRIS: Biomedical Translational Research Information System
CBPR: Community-based participatory research
CONSORT: Consolidated Standards of Reporting Trials
COVID-19: Coronavirus disease-2019
D.C. CHOC: Cardiovascular Health and Obesity Collaborative
DC COOKS with Heart: DC Community Organizing for Optimal Culinary Knowledge study with Heart
IRB: Institutional Review Board
IT: Information technology
NIH: National Institutes of Health
REDCap: Research Electronic Data Capture
SARS-CoV-2: Severe Acute Respiratory Syndrome Coronavirus
SMS: Short Message Service
