# Supplementary material for: Going virtual during the COVID-19 pandemic: adaptation of a mixed-methods dietary behavior study within a community-based participatory research study of African-American adults at risk for cardiovascular disease
Source: BMC Med Res Methodol. 2022 Dec 22;22:330. doi: 10.1186/s12874-022-01806-3 (PMC9773576; doi:10.1186/s12874-022-01806-3)
Supplement: Supplementary file 1 — Additional file 1. Virtual focus group moderator’s guide for DC COOKS (2021). [file 12874_2022_1806_MOESM1_ESM.docx]

VIRTUAL focus GROUP MODERATOR’S GUIDE

FOR

DC COOKS (2021)

A. Introduction

1. Welcome, and thank you for joining. Your participation here today is essential for the success of an intervention study we plan to undertake.
2. Introduce yourself as the moderator, and then follow by introducing the other co-facilitator. Introduce other team members and their roles.

- (Describe the role of the moderator and the facilitators and each team member)

1. Allow each of the focus group members the opportunity to introduce themselves. Ice breaker – share screen with pictures/names of different food options
2. Briefly describe the focus group as similar to an opinion survey, but with more general and broad questions.

“A focus group is a special type of group in terms of purpose, size, composition, and procedures. It is a way to better understand how people think about an issue, product, or service. You were asked to participate in this focus group because each of you are a member of the community that we are examining in our main study. So, we are trying to get feedback on study questionnaires and intervention in the main study used for this research project.”

Moderator Introduction of Format of Focus Group:

*My role is to coordinate the discussion. The work that needs to be done here is dependent on your full participation.*

*As I mentioned before, _________________ (moderator /co-facilitator) and _________________ is the co-facilitator. We will be taking notes and using the tape recorder with the assistance of our other team members to make sure we don’t forget your important ideas.*

For the purpose of this focus group, we want to test and consider from the point-of-view of potential study participants and community members, thoughts on a larger research study that aims to provide cooking and meal preparation lessons to improve heart health and reduce obesity in Washington DC.

*Opinions and ideas that are expressed in this room should stay in this room. We will need to respect one another’s right to confidentiality. By confidentiality, I mean that nothing that is said within this group today should be discussed with anyone outside this room. Also, no one would tell your identity, nor could anyone figure who you are in our study. Although we are tape recording this session, only first names will be used to assist us in transcribing (typing up) the key ideas that are raised. When results from our focus groups are discussed at professional meetings or published in medical journals, no personal identifiers (no full names) will be used)*

*If something I am asking you is hard to understand, please signal one of us so we can assist you. This will only help us do a better job in our research. If it’s challenging for one person it likely is confusing for someone else too.*

*In order to minimize multiple conversations, we would like to ask for you to use the hand raise function then wait for one of us to call on you. We will allow everyone a certain amount of time to speak up if you have a comment and something to say. We encourage open discussion, but you will need to speak one at a time. Since what each of you will have to say is extremely important in this process, it is necessary for us to accurately record what you have to say.*

*We only have a limited amount of time to complete this process, and therefore we must stay on-track. If I, as the moderator, change the direction of the discussion, or have to stop someone from continuing with what they are saying, it will only be due to time considerations, and should not be taken personally. We are thankful that you have agreed to participate in this focus group, and I will do what I can so that you get done in a timely manner.*

*Your opinions and perspectives are necessary for this process. Your complete honesty as to what sorts of issues* ***you or others within your community*** *may face regarding food, food options, or cooking are extremely necessary. We are interested in all your ideas, comments, and suggestions.* ***There are no “right” or “wrong” answers****. All of your comments, both positive and negative, are helpful. Don’t worry about offending us with negative comments. We are here to learn from you.*

**II. Group Discussion/ Interviewing Session**

- 1. **Questionnaires Follow-up**
  2. **Cooking and meal preparation – lived experience questions**
  3. **Questions about study plan, curriculum, recipes, lesson videos**

1. Questionnaires

Moderator can refer participants to use paper copies of the survey questions if they need.

1. Were there any questions that were difficult to understand or not very clear? If so, please tell me the question number on the survey that you identify as being “difficult”? Moderator may use list of measures that team thinks may have been difficult or that team saw as having long response times.
2. What are your thoughts regarding the length of the questionnaires?
3. Is it possible that any of the items may be considered too personal, or too offensive to answer?
4. What suggestions do you have for improving the questionnaires?
5. Were there any questions regarding beliefs or behaviors related to cooking and meal preparation that were left out that you would like to see included?
6. What do you see as potential barriers to filling out the questionnaires? Moderator may use these prompts: technological, language of questions, time

**B. Cooking and meal preparation – lived experience questions**

Now, that you have provided input on the questionnaires I would like to ask you a few general questions about your cooking and meal preparation practices. If you do not cook routinely, think about the times when you may have helped others cook or prepare a meal.

1. What gets in the way of you cooking? Of going shopping for food? Enter probes – time, work hours, location of stores to purchase food, your personal or family preferences, cost, fear/concern of getting the COVID infection, social distancing/mandatory mask use regulations, any other issues due to current pandemic situation
2. What motivates you to cook?
3. Tell me about your experiences with shopping for food in your neighborhood or in stores in which you shop that may not be in your neighborhood?
4. Have participants start with a meal that they like to cook or enjoy having someone cook/prepare for them and then ask: When you cook or prepare food, describe how you are feeling and what you are thinking when you are cooking in as much detail as possible. Describe in your own words what you are thinking or concentrating on during the process of preparing the meal. For example are you paying attention to the smell or color of the food?

1. Do you have any rituals or practices that you do when cooking? Or at the start or during a meal, do you have any rituals? Moderator may use these as prompts: prayer, moment of appreciation, mindful moment, pictures
2. Are there any other things about your experiences regarding cooking that we haven’t discussed but you would like to share?

**C. Questions about study plan, curriculum, recipes, lesson videos**

Moderator can refer participants to use overview slide of study if they need.

Now, we would like to get your thoughts on our team conducting a cooking study in this community. The purpose of the study is to evaluate cooking and food shopping behavior following a series of cooking lessons using recipes geared towards healthy, yet flavorful foods. The study will consist of 6 weekly lessons that are 2 hours long provided by a local chef. The lessons will occur on a weekday (late morning or afternoon) and will include participating in a group of 3-4 people to prepare a recipe selected by the chefs, each group will be instructed by the chef and one to two medical students trained in culinary medicine. Each lesson will focus on an aspect of general nutrition- example explaining different types of carbohydrates or fats. After preparing each recipe, all groups will sit down to a shared meal with the chef. Prior to the start of the lessons, there will be a visit at the Clinical Center in Bethesda, MD to take surveys, report on home food intake to a dietician, have a cooking assessment, and laboratory blood work done. This type of visit will also occur 6 weeks after the last cooking lesson and then again 12 weeks after the last cooking lesson. Throughout the entire study time period of 18 weeks (3 months), participants will be asked to complete a paper study diary to report their home cooking and meal preparation habits. For each visit of the study, participants will be compensated and will have transportation through taxi cab to and from their home and the NIH Clinical Center. We planned this study prior to the start of the COVID pandemic. There might be some changes as required due to COVID pandemic, however we will make sure to follow all the mandatory guidelines as participants safety is our prime concern. We appreciate any thoughts you have on conducting this study during the pandemic or shortly after the pandemic ends. As we go through specific questions about the study, you may share your thoughts, if any, concerning the pandemic.

1. What thought, if any, do you have about the purpose of the study?
2. What thoughts, if any, do you have about having the study occur in this community (neighborhood)?

Moderator prompts: Add prompts about delivery of this study virtually or in person or both. What will be barriers for either – e.g. in person – risk of transmission; virtual – technology in the home, family members

1. What motivators (possible reasons) could exist for people to participate in this study? Enrollment and staying in the study for the entire time.
2. What barriers (issues) could exist to prevent participation in this study? Enrollment and staying in the study for the entire time.

Show (share screen) samples of cooking journal pages?

1. What thoughts do you have about the cooking journal pages? Would the questions on the page capture your cooking habits, patterns?

Show (share screen) overview of structure of each lesson, then show video from a lesson or video from chef instructor (2 min)

1. Are there any thoughts about the lesson – content, foods selected to discuss? Do you think the topic is/could be helpful to someone – Probe: helpful in terms of something they want to learn, helpful to their health, helpful for them to know for others

Show (share screen) examples of recipes from same lesson

1. Are there any thoughts on the layout of the recipe –Probe: Is it easy to understand? Easy to read and visualize what steps you have to do?
2. Would you make this recipe at home, would you recommend that a family or friend make it (based on just reading the recipe)? And why? Probe: do you think it will be enjoyable, flavorful, easy or quick to prepare?
3. Based on what you have seen, what (if anything) would you change about the recipes?
4. Based on what we have described about the study, what if anything would you change?
5. What would be one piece of advice that you would give the research team before beginning the study?

Closing:

- After the final question, the moderator would thank the participants for taking part of this focus group
- The moderator and principal investigator (if present) will express their appreciation for the comments and the information exchange from the participants of the focus group.
- Moderator will inform the participants that their feedback will be a part of a process of designing the best way to administer the study questionnaire to participants.
- The moderator will remind the participants about confidentiality in discussion of this study with others.
- Moderator reviews with participants process for receiving compensation.
- Moderator thanks participants for attending.
